# lncRNA and Mechanisms of Drug Resistance in Cancers of the Genitourinary System

**DOI:** 10.3390/cancers12082148

**Published:** 2020-08-03

**Authors:** Dominik A. Barth, Jaroslav Juracek, Ondrej Slaby, Martin Pichler, George A. Calin

**Affiliations:** 1 Research Unit of Non-Coding RNAs and Genome Editing in Cancer, Division of Clinical Oncology, Department of Medicine, Comprehensive Cancer Center Graz, Medical University of Graz, 8036 Graz, Austria; dominik.barth@medunigraz.at (D.A.B.); martin.pichler@medunigraz.at (M.P.); 2 Department of Translational Molecular Pathology, The University of Texas MD Anderson Cancer Center, Houston, TX 77030, USA; juracekjaroslav@gmail.com; 3 Department of Comprehensive Cancer Care, Masaryk Memorial Cancer Institute, 62500 Brno, Czech Republic; on.slaby@gmail.com; 4 Central European Institute of Technology, Masaryk University, 62500 Brno, Czech Republic; 5 Department of Experimental Therapeutics, The University of Texas MD Anderson Cancer Center, Houston, TX 77030, USA

**Keywords:** lncRNA, drug resistance, chemoresistance, prostate cancer, renal cell carcinoma, bladder cancer, seminoma

## Abstract

Available systemic treatment options for cancers of the genitourinary system have experienced great progress in the last decade. However, a large proportion of patients eventually develop resistance to treatment, resulting in disease progression and shorter overall survival. Biomarkers indicating the increasing resistance to cancer therapies are yet to enter clinical routine. Long non-coding RNAs (lncRNA) are non-protein coding RNA transcripts longer than 200 nucleotides that exert multiple types of regulatory functions of all known cellular processes. Increasing evidence supports the role of lncRNAs in cancer development and progression. Additionally, their involvement in the development of drug resistance across various cancer entities, including genitourinary malignancies, are starting to be discovered. Consequently, lncRNAs have been suggested as factors in novel therapeutic strategies to overcome drug resistance in cancer. In this review, the existing evidences on lncRNAs and their involvement in mechanisms of drug resistance in cancers of the genitourinary system, including renal cell carcinoma, bladder cancer, prostate cancer, and testicular cancer, will be highlighted and discussed to facilitate and encourage further research in this field. We summarize a significant number of lncRNAs with proposed pathways in drug resistance and available reported studies.

## 1. Introduction

Cancers of the genitourinary system, including renal cell carcinoma (RCC), bladder cancer (BC), prostate cancer (PCa), and testicular cancer (TC) add up to being responsible for 626,000 cancer related deaths worldwide each year. Together they account for about 14% of all malignancies, respectively [1]. While TC has a comparably good prognosis even in metastatic treatment settings, outcomes for RCC, BC, and PCa strongly vary depending on tumor stage and clinico-pathological biomarkers [2]. However, because of the introduction and availability of novel drugs such as second generation antiandrogens in castration resistant PCa (CRPC) [3] or immune checkpoint inhibitors in BC and RCC [4,5], outcomes are improving. Yet, resistance to systemic cancer therapy is a great obstacle in cancer treatment and represents a complex process involving genetic and epigenetic mechanisms. Insensitivity to systemic cancer treatment may be divided into intrinsic, i.e., resistance is present before any treatment, and acquired resistance by selection pressure [6].

Long non-coding RNAs (lncRNA) are non-protein coding RNA molecules with a length of more than 200 nucleotides and exert regulatory function in various cellular processes [7,8,9,10,11,12]. Briefly, a lncRNA may act as a signal, guide, decoy or scaffold for other non-coding RNAs or proteins and thereby alter various cellular functions [13]. In cancer, lncRNAs can act as both tumor suppressors and oncogenes and their involvement in cancer development and progression, including in genitourinary cancers, was repeatedly demonstrated [14].

In this review, we give a comprehensive overview of the existing evidence on the mechanisms of drug resistance involving lncRNAs in cancers of the genitourinary system as they may represent future therapeutic targets (Table 1).

## 2. Long Non-Coding RNAs and Drug Resistance in Renal Cell Carcinoma

According to the GLOBOCAN database, kidney cancer accounts for 338,000 of cancer diagnoses worldwide each year [1], making it the second and third most common cancer of the urogenital system in men and women, respectively. Ninety percent of malignancies of the kidney account for RCC, which is derived from the epithelium of renal tubules [49]. The most frequent pathological subtypes are clear cell (ccRCC), papillary RCC, and chromophobe RCC, which account for 70–80%, 10–15%, and 3–5%, respectively [50]. This traditional classification has become far more complex as RCCs can display various histological features of other subtypes and these novel molecular classifications bring new perspectives [51]. For localized disease, surgery represents the treatment of choice. However, some patients eventually experience recurrence and develop metastasis or already present with metastatic dissemination at the time of diagnosis. In these cases, palliative treatment systemic therapy is indicated. As RCC shows insufficient response to chemotherapy [52], immunotherapy with interleukin-2 and IFNα were some of the first agents used in the systemic treatment of RCC with only moderate success [53]. Novel targeted therapies such as the multikinase inhibitors sunitinib and sorafenib, which target vascular endothelial growth factor receptor (VEGFR) and platelet derived growth factor receptor (PDGFR) thereby inhibiting angiogenesis, improved these outcomes [54,55]. Nonetheless, the introduction of immune checkpoint inhibitors such as nivolumab, pembrolizumab and combinations with antiangiogenetic agents in the treatment of ccRCC drastically improved the outcome of metastatic RCC [5,56]. However, a significant number of patients still do not benefit from these novel therapeutic concepts and currently available predictive biomarkers, such as PD-L1 expression perform insufficiently [57]. Therefore, understanding the mechanisms of drug resistance is crucial. Importantly, intratumor heterogeneity plays an important role in treatment failures in RCC patients [58,59]. In RCC, lncRNAs were described to engage in pathogenesis and disease progression and were suggested as novel diagnostic tools [60]. However, although lncRNAs participate in the regulation of checkpoint inhibitors, studies investigating the development of resistance to checkpoint inhibitors in RCC are lacking [61,62]. To date, only data on lncRNAs and their role in in the development of resistance to multikinase inhibitors and chemotherapy are available.

### 2.1. lncRNAs Promoting Drug Resistance in RCC

#### 2.1.1. SRLR

**Figure 1:**
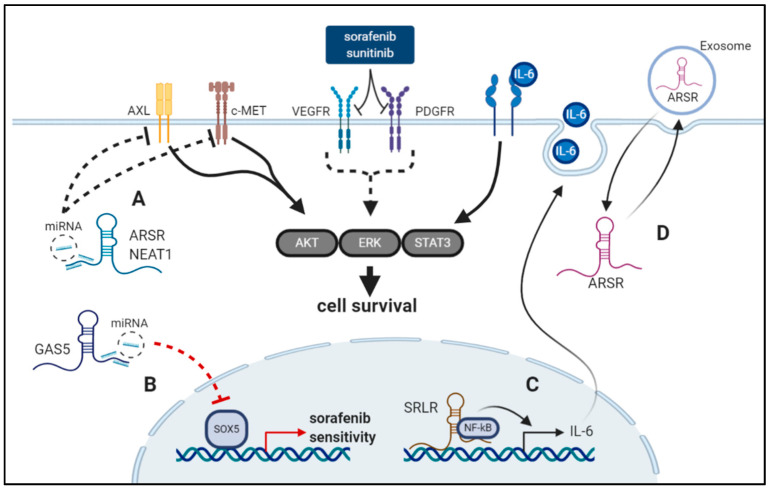
Examples of long non-coding RNAs (lncRNAs) and mechanisms of drug resistance in renal cell carcinoma (RCC). (**A**) lncRNA Nuclear Paraspeckle Assembly Transcript 1 (NEAT1) sponges miR-34a and as a result, increases c-MET expression. (**B**) lncRNA growth arrest specific transcript 1 (GAS5) sponges miR-21 thereby preventing it from targeting SRY-Box transcription factor 5 (SOX5) leading to increased sensitivity to sorafenib. (**C**) lncRNA sorafenib resistance-associated lncRNA in RCC (SRLR) recruits NF-κB to the interleukin 6 (IL-6) promoter, resulting in increased autocrine IL-6 secretion and bypassing of blocked vascular endothelial growth factor receptor (VEGFR) and platelet derived growth factor receptor (PDGFR). (**D**) lncRNA activated in RCC with sunitinib resistance (ARSR) can be secreted via exosomes from sunitinib resistant cells and can be incorporated from sunitinib sensitive cells, thereby transferring drug resistance. ARSR sponges miRNAs and consequently promotes AXL and c-MET expression promoting sunitinib resistance. (created with Biorender.com).

The sorafenib resistance-associated lncRNA in RCC (SRLR) was first functionally investigated by Xu et al. [15] who reported SRLR to promote resistance to treatment with the multi-kinase inhibitor sorafenib. Overexpression of SRLR was observed in RCC cells and the tissue of patients that were resistant to sorafenib. Mechanistically, SRLR directly interacts with the transcription factor NF-_Κ_B which subsequently activates interleukin-6 (IL-6) transcription and autocrine IL-6 secretion in RCC cells. This results in the activation of the STAT3 pathway and sidesteps the inhibition of receptor tyrosine kinases such as VEGFR and PDGFR by sorafenib (Figure 1). This was experimentally confirmed both in vitro and in vivo [15]. The relationship of SLRL and IL-6 was only recently confirmed in a study investigating SRLR in polycystic ovary syndrome [63]. In a clinical dataset that included 95 RCC patients, higher expression levels of lncRNA SRLR were associated with reduced progression-free survival (PFS) (HR = 0.407, 95%CI = 0.222–0.744, *p* = 0.003) and was additionally related to low benefit to treatment with sorafenib, and high levels of IL-6 [15].

#### 2.1.2. ARSR

The lncRNA activated in RCC with sunitinib resistance (ARSR) significantly impacts resistance to treatment with the multikinase inhibitor sunitinib in RCC. [16]. Mechanistically, by acting as a competing endogenous RNA (ceRNA), ARSR sequesters miR-34 and miR-449 and thus increases the levels of their targets AXL and c-MET, thereby promoting sunitinib resistance (Figure 1). ARSR is overexpressed in sunitinib resistant cells and reciprocally ARSR expression is increased by AXL through activation of FOXO transcriptional factors. This suggests a positive feedback loop between AXL and ARSR in sunitinib-resistant RCC. Interestingly, sunitinib resistance may also be transferred from cells resistant to sunitinib to sunitinib-sensitive cells via exosome-mediated transmission. Targeting of ARSR could be used as a novel therapeutic approach to overcome sunitinib resistance, as shown in in vivo and in vitro experiments [16]. Moreover, pretreatment ARSR levels in the plasma of RCC patients is significantly correlated with poor PFS for high vs. low ARSR expression (HR = 2.9, 95%CI = 1.2–7.1, *p* = 0.017), corroborating these findings [16]. A recent study also proposed single nucleotide polymorphisms (SNP) of the ARSR sequence as potential biomarkers for RCC outcome [64].

#### 2.1.3. NEAT1

The nuclear paraspeckle assembly transcript 1 (NEAT1) and its function as an oncogenic lncRNA is already well investigated in multiple studies [65]. NEAT1 has been reported to promote resistance to chemotherapy [66,67]. In RRC, NEAT1 may inhibit response to sorafenib treatment by regulation of the NEAT1/miR-34a/c-MET axis by acting as a sponge for miR-34a [18] (Figure 1). Both, c-MET and miR-34a were already reported to impact chemoresistance in other cancer entities such as esophageal cancer and osteosarcoma [68,69,70]. Moreover, NEAT1 shows high expression in RCC cell lines and tissues. Additionally, overexpression of NEAT1 was correlated with epithelial–mesenchymal transition (EMT) and also significantly correlated with poor OS and PFS in RCC; although no uni- and multivariate analyses were implemented in the study by Liu et al., along with no xenograft models [18].

### 2.2. lncRNAs Enhancing Drug Sensitivity in RCC

#### 2.2.1. ADAMTS9-AS2

The lncRNA ADAMTS9 antisense RNA 2 (ADAMTS9-AS2) was already reported to influence drug resistance in cancer. However, its role may vary depending on the cancer type as downregulation of ADAMTS9-AS2 in breast cancer was reported to increase tamoxifen resistance, whereas its downregulation was associated with increased sensitivity to temozolomide in glioblastoma [71,72]. In RCC, ADAMTS9-AS2 is downregulated and high expression is significantly associated with better OS [17]. By sequestering miR-27-3p, overexpression of ADAMTS9-AS2 resulted in increased FOXO1 expression and restored chemosensitivity to 5-fluorouracil and cisplatin. However, confirmation in in vivo experiments is missing [17]. To date, chemotherapy is not a valid treatment option in RCC as it has proven ineffective, therefore the direct clinical impact of the study is limited [52]. However, targeting lncRNAs may overcome chemoresistance in RCC in the future and may establish chemotherapy as a valid treatment option in RCC.

#### 2.2.2. GAS5

The lncRNA growth arrest specific transcript 1 (GAS5) was reported to influence RCC resistance to sorafenib in a study by Liu and colleagues [19]. The tumor suppressive role of GAS5 in RCC carcinogenesis and progression has already been repeatedly demonstrated [73]. In terms of its impact on sorafenib resistance, it was shown to act as a sponge for miR-21 and upregulation of GAS5 resulted in likewise upregulation of the transcription factor sex determining region Y-box protein 5 (SOX5), conferring increased sensitivity to sorafenib [19]. This was demonstrated by multiple in vitro and in vivo models. All effectors in the GAS5/miR-21/SOX5 pathway as proposed by Liu et al. [19] have already been reported to be effectors in chemoresistance individually, which corroborates these results [74,75,76,77].

## 3. lncRNAs and Drug Resistance in Bladder Cancer

With approximately 550,000 new cases in 2018, BC represents the seventh most frequent tumor type in our population [78]. The most common histological type is urothelial carcinoma (UC), accounting for approximately 90% of all bladder cancers. Other less frequent subtypes are squamous cell carcinoma and adenocarcinoma [79]. Based on the TNM classification and histopathological grading, two major subtypes of UC—non-muscle-invasive carcinoma (NMIBC; around 80% at diagnosis) and muscle-invasive carcinoma (MIBC) are distinguished [80].

NMIBC is a heterogeneous disease with a good prognosis and curability, nevertheless around 70% of cases recur and about 10% progress into an invasive phenotype [81]. The standard-of-care for NMIBC currently is transurethral resection of bladder tumor (TURBT) with optional addition of intravesical therapy (mitomycin C, doxorubicin, gemcitabine, or BCG—Bacillus Calmette-Guerin) [82]. In the case of MIBC, the standard treatment protocol involves radical cystectomy with neoadjuvant or adjuvant chemotherapy with gemcitabine/cisplatin. Also, in metastatic disease which develops in 50% of MIBC cases, cisplatin-based chemotherapy in combination with gemcitabine remains the main therapeutic modality [83]. Moreover, novel treatment options such as immune checkpoint and fibroblast growth factor receptor (FGFR) inhibitors were introduced [4,84]. Despite recent advances in BC systemic treatment, eventual treatment resistance is responsible for cancer progression and death and its molecular mechanisms remain rather unclear. LncRNAs play a major part in BC pathophysiology [14,85,86] and therefore are suggested as essential molecules in drug resistance. Further research of lncRNA involvement in BC chemoresistance may thus improve current treatment and reveal new therapeutic targets in BC.

### 3.1. lncRNAs Promoting Drug Resistance in BC

#### 3.1.1. UCA1

**Figure 2:**
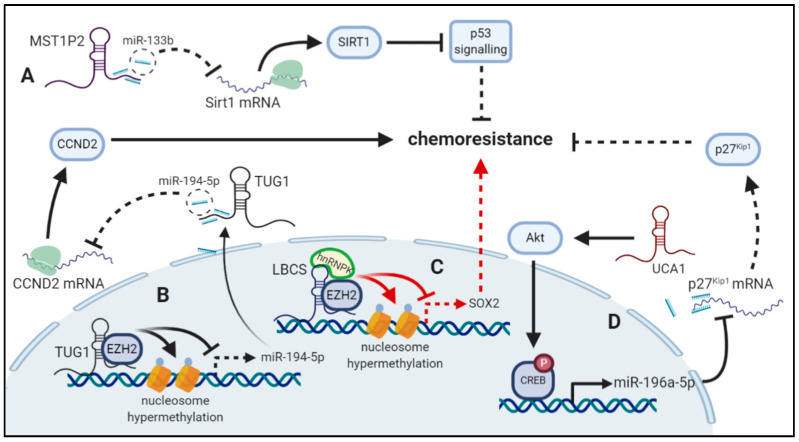
Examples of long non-coding RNAs (lncRNAs) and mechanisms of drug resistance in bladder cancer (BC). (**A**) lncRNA macrophage stimulating 1 pseudogene 2 (MST1P) sponges miR-133b thereby preventing it from targeting sirtuin 1 (Sirt1). This leads to increased Sirt1 expression and inhibition of p53 by Sirt1 and thus increased chemoresistance. (**B**) lncRNA taurine up-regulated 1 (TUG1) recruits enhancer of zeste homolog 2 (EZH2) to the miR-194-5p promoter resulting in hypermethylation and thus inhibited miR-194-5p transcription. Additionally, TUG1 sponges miR-194-5p preventing it from targeting cyclin D2 (CCND2) leading to increased expression and enhanced chemoresistance. (**C**) lncRNA low expressed in bladder cancer stem cells (LBCS) recruits EZH2 and heterogeneous nuclear ribonucleoprotein K (hnRNPK) to the SRY-box transcription factor 2 (SOX2) leading to hypermethylation and thereby inhibiting chemoresistance. (**D**) lncRNA urothelial cancer associated 1 (UCA1) activates the Akt-pathway and thereby enhances phosphorylation of cAMP response element-binding protein (CREB) transcription factor. CREB promotes the transcription of miR-196a-5p which targets p27Kip1 tumor suppressor, leading to enhanced chemoresistance by UCA1 upregulation. (created with Biorender.com).

The lncRNA urothelial cancer-associated 1 (UCA1) plays an important role in BC tumorigenesis as shown by increased proliferation, invasion, migration, as well as therapy resistance of UC cell lines [86]. Regarding cisplatin chemoresistance, high expression of UCA1 in resistant cells significantly increases cell viability during cisplatin treatment. Moreover, overexpression positively regulates expression of Wnt6, subsequently activating Wnt signaling [21], which was previously connected with chemoresistance in cancer [87]. In addition to cisplatin, UCA1 was also studied in relation to gemcitabine resistance, where functioning via the UCA1/CREB/miR-196a-5p axis is proposed [20]. In this paradigm, UCA1 activates the AKT signaling pathway, which results in proto-oncogenic transcription factor CREB (cAMP response element-binding protein) phosphorylation. Active CREB then positively regulates expression of oncogenic miR-196a-5p (Figure 2) [20]. It has been shown that the suppression of miR-196a could attenuate resistance to cisplatin in lung cancer cell lines and relates to drug efflux-related proteins such as multidrug resistance1 (MDR1), multidrug resistance associated protein 1 (MRP1), endonuclease non-catalytic subunit (ERCC1), survivin, or Bcl-2 [88]. However, in BC, miR-196a-5p controls p27^Kip1^ expression by directly binding to its 3′UTR [20]. As a cyclin-dependent kinase (CDK) inhibitor and activator of cleaved-caspase 3, p27^Kip1^ can promote drug resistance via cell apoptosis [89]. Most recent reports showed a new regulatory network involving miR-582-5p and ATG7-mediated autophagy inhibition [22]. It is presumed that UCA1 serves as a miRNA sponge and binds mature miR-582-5p. Among miR-582-5p direct targets is also ATG7—an E1-like activating enzyme involved in autophagy [90], which is often activated as a protective mechanism of resistant cancer cells during chemotherapy [91]. Indeed, miR-582-5p-mediated suppression of ATG7 could inhibit autophagy, indicating the UCA1/miR-582-5p/ATG7 pathway regulates BC anti-cancer drug response [22].

#### 3.1.2. TUG1

Taurine-upregulated gene 1 (TUG1) is a lncRNA initially identified in the development of retina [92]. Recent findings have proven its association with various cancers; dysregulated expression level of TUG1 was described in colorectal cancer [93], gastric cancer [94], non-small cell lung cancer (NSCLC) [95], hepatocellular cancer [96], and BC [85,97]. Mechanisms enabling TUG1 to regulate cellular processes including chemoresistance were, for instance, described in lung cancer [95] though its biological function in BC is still unclear. Yu et al. [23] showed that TUG1 can induce the chemoresistance to cisplatin in BC through the TUG1/miR-194-5p/CCND2 axis. In detail, TUG1 influences miR-194-5p level via sponging mature miR-194-5p molecules and enhancer of zeste homolog 2 (EZH2)-related promoter methylation. Both ways lead to miR-194-5p down-regulation resulting in higher levels of its direct target cyclinD2 (CCND2) and thus chemoresistance to cisplatin promotion (Figure 2). Interestingly, this regulatory mechanism impacts proliferation and apoptosis of BC as well [23].

#### 3.1.3. PVT1

Another chemoresistance-related lncRNA is plasmacytoma variant translocation 1 (PVT1). This 1716 nt long transcript is overexpressed in various human cancers [98,99,100] and its regulative effect on chemosensitivity was already closely described in cervical cancer [98], gastric cancer [99] and lung cancer [100]. In BC, PVT1 overexpression was confirmed in chemo-resistant tissues, where it negatively correlates with response to cisplatin and doxorubicin. Also, in T24/DR cell lines, PVT1 knockdown reduced resistance to cisplatin and doxorubicin and led to suppression of MDR1 and MRP1 expression. Moreover, PVT1 suppression inhibits Wnt/β-catenin signaling probably via miR-200b which is epigenetically silenced by PVT1. This proposed mechanism is supported by the observed restoration of chemoresistance in PVT1 knockdown T24/DR cells after β-catenin upregulation [24].

#### 3.1.4. FOXD2-AS1

FOXD2 adjacent opposite strand RNA 1 (FOXD2-AS1) is an oncogenic cancer-related lncRNA, which was reported to be overexpressed in BC and was connected to tumor stage, recurrence as well as poor prognosis. FOXD2-AS1 promotes BC cell proliferation, migration, and invasion mainly via regulation of Tribbles pseudokinase 3 (TRIB3), which negatively regulates Akt [101]. Recently, a study by An et al. [25] described the involvement of FOXD2-AS1 in gemcitabine resistance of BC. In gemcitabine-resistant BC cells, a high level of this lncRNA led to the upregulation of known genes that are associated with drug resistance, such as MDR1, MRP2, LDL receptor-related protein 1 (LRP1), or ATP binding cassette subfamily C member 3 (ABCC3) protein [25]. The ABCC3 protein is demonstrably increased in BC cells and enhances cell proliferation, drug resistance, and aerobic glycolysis [102]. The bioinformatic analysis in this study then identified miR-143 as the intersection between FOXD2-AS1 and ABCC3, as miR-143 is predicted to target the ABCC3 3′-UTR and at the same time matched with FOXD2-AS13′-UTRs. Indeed, FOXD2-AS1 acted as a ceRNA miR-143 and thereby increased ABCC3 protein expression and accelerated gemcitabine resistance [25].

#### 3.1.5. DLEU1

The deleted in lymphocytic leukemia 1 lncRNA (DLEU1) was discovered due to its location on chromosome 13q14.3, a region which is commonly deleted in chronic B-cell lymphocytic leukemia [103,104,105]. Beside DLEU1, this locus also hosts miR-15a and miR-16 which are the first ever discovered ncRNAs to be involved in human diseases [106,107,108,109]. Though in hematopoietic tumors DLEU1 can act as a potential tumor suppressor [110], in other cancers including cervical cancer [111], colorectal cancer [112], and NSCLC [113] it has been shown to exert oncogenic function. In BC, DLEU1 is significantly increased and induces cell proliferation, invasion, and cisplatin resistance. The mechanism involves sponging miR-99b [26], which was described as a tumor suppressive factor in multiple cancers [114,115]. The same function is suggested in BC where miR-99b shows significant downregulation [26] and targets, among other genes, FGFR3 [115] which is constitutively activated in NMIBC [116]. Sponging miR-99b by DLEU1 results in increased expression of oncogenic membrane protein HS3ST3B1 [26]. Although limited information about the exact function in BC is known, in other tumors HS3ST3B1 is associated mainly with EMT [117].

#### 3.1.6. MST1P2

Association with resistance to cisplatin in BC was documented for macrophage stimulating 1 pseudogene 2 (MST1P2). MST1P2 was reported to be significantly upregulated in cisplatin-resistant BC cell lines where it serves as a ceRNA. Indeed, MST1P2 sponges miR-133b, where the expression level was downregulated in the same cell lines [27]. Interestingly, the participation of miR-133b in the regulation of chemoresistance was already described in ovarian cancer [118], colorectal cancer [119], and osteosarcoma [120]. Among direct targets of this short RNA is oncogene Sirt1 (Sirtuin 1) which was proposed as a possible effector of MST1P2/miR-133b mediated drug resistance [27]. Sirt1 overexpression can inactivate p53, which results in a low response of cancer cells with unmutated p53 to DNA-damaging chemotherapeutics [121] and thus inhibition of cancer cells apoptosis (Figure 2).

#### 3.1.7. HIF1A-AS2

The hypoxia-inducible factor-1 alpha antisense RNA-2 (HIF1A-AS2) is a natural antisense transcript of hypoxia-inducible factor-1alpha (HIF-1α) which was suggested to play a crucial part in tumorigenesis, mainly via regulation of the HIF-1α pathway [122]. In BC, HIF1A-AS2 displays an oncogenic function since it promotes cell proliferation, migration, and suppresses apoptosis [123]—however, the exact molecular regulatory mechanisms are yet to be elucidated. Interestingly, HIF1A-AS2 is highly upregulated also in cisplatin-resistant BC cells and tissues and contributes to BC cisplatin chemoresistance [28]. Previous reports in glioblastoma showed that HIF1A-AS2 interacts with proteins IGF2BP2 and DHX9 while enhancing the expression of their targets such as high mobility group AT-hook 1 (HMGA1) [124]. A similar mechanism takes place in BC drug resistance where HMGA1 overexpression facilitated by HIF1A-AS2 inhibits the transcriptional activity of p53 family proteins leading to cisplatin-induced apoptosis restraint [28].

#### 3.1.8. GHET1

The oncogenic lncRNA gastric carcinoma proliferation-enhancing transcript 1 (GHET1) is upregulated in BC tissues, where its level is associated with tumor size, higher tumor stage, lymph node involvement, and adverse prognosis [125]. A recent report showed that GHET1 is also related to sensitivity to gemcitabine in BC. Based on statistics using TCGA (The Cancer Genome Atlas) datasets, a high level of GHET1 was correlated with increased expression of the ABCC1 gene, which is related to multidrug resistance [29]. This protein was already associated with chemotherapeutic resistance in BC [126], nevertheless, the exact mechanism of GHET1 regulation of the expression of ABCC1 is still unknown. However, it is presumed that the underlying mechanism involves regulation of miRNAs targeting ABCC1 as described in glioma, where GHET1 promoted a malignant phenotype through down-regulation of miR-216a [127].

#### 3.1.9. MALAT1

Another lncRNA mediating cisplatin resistance in BC is metastasis-associated lung adenocarcinoma transcript 1 (MALAT1). Liu et al. [30] showed that this function is based on the regulation of the miR-101-3p/VEGF-C pathway. In detail, direct interaction between MALAT1 and miR-101-3p, which was demonstrated to be a tumor suppressor [128], impairs targeting and leads to VEGF-C overexpression [30]. This cytokine has been earlier described as a possible effector in BC chemoresistance via regulation of a mammary serine protease inhibitor maspin [129]. In addition, miR-101-3p reportedly enhances sensitivity of BC to cisplatin also by targeted silencing of EZH2 and MRP1 expression [130].

### 3.2. lncRNAs Enhancing Drug Sensitivity in BC

#### 3.2.1. lncRNA-LET

The lncRNA-low expression in tumor (lncRNA-LET) was suggested to be involved in drug response in BC. As shown in the study of Zhuang et al. [31], gemcitabine treatment of chemoresistant urothelial cancer cells led to lncRNA-LET down-regulation and to the enrichment of cancer stem-like cell population. Subsequent deregulation of the lncRNA-LET/NF90/miR-145 pathway promoted stemness of cancer cells and led to chemoresistance. This complex mechanism is initiated by gemcitabine treatment-related upregulation of TGFβ1. Activation of TGFβ/SMAD signaling by SMAD binding element (SBE) in the lncRNA-LET promoter represses this lncRNA which results in greater stability of the NF90 protein [31]. Nuclear factor 90 (NF90) is a double-stranded RNA-binding protein participating in various cellular processes such as transcription, translation, or mRNA stabilization [131]. Interestingly, NF90 was also described as an adverse regulator in the miRNA processing pathway [132]. Indeed, in this case NF90 acts as competitor for the association of the microprocessor complex with pri-miR-145 resulting in miR-145 biogenesis inhibition. Subsequently, the downregulation of miR-145 leads to higher expression of cancer stemness regulatory genes Krüppel-like factor 4 (KLF4) and high-mobility group AT-hook 2 (HMGA2) [31] and thus chemoresistance. Direct regulation of KLF4 with miR-145 was reported previously in connection with the vascular smooth muscle cell phenotype [133].

#### 3.2.2. GAS5

GAS5 participates in chemotherapeutic resistance to doxorubicin in urothelial carcinoma, although the underlying mechanism remains unclear. In BC cells resistant to doxorubicin, the GAS5 expression level was shown to be negatively correlated with the chemotherapy resistance to this drug [32]. Moreover, GAS5 overexpression positively correlated with apoptosis induced by doxorubicin treatment, as the repressed expression of anti-apoptotic protein Bcl-2 was observed. Inversely, upregulation of Bcl-2 reversed the inhibitory effect of GAS5 on chemoresistance to doxorubicin in chemo resistant cells [32]. This is no surprise, as GAS5 is notably known for apoptosis promotion, mainly via Bcl-2 suppression and caspase-3 upregulation, which is possibly facilitated through the miR-155 regulatory pathway [134].

#### 3.2.3. LBCS

The lncRNA LBCS (low expressed in bladder cancer stem cells) acts as an important tumor suppressor in bladder cancer stem cells (BCSC) self-renewal and chemoresistance [33]. In addition to downregulation in BC, its level associates with tumor grade, response to chemotherapy treatment, and prognosis. The chemoresistance suppression is executed by guiding the hnRNPK–EZH2 complex [33]. Heterogeneous nuclear ribonucleoprotein K (hnRNPK) is an important cancer-related RNA- and DNA-binding protein associated with poor prognosis in BC [135], while EZH2 is a histone methyltransferase that acts as an oncogene important for self-renewal [136]. As mentioned before, LBCS acts as a scaffold, thereby facilitating the formation of the hnRNPK/EZH2 complex. Subsequent recruitment of the complex to the SOX2 promoter mediates H3K27me3 and leads to SOX2 suppression [33]. A low level of LBCS therefore contributes to the upregulation of SOX2, a previously confirmed marker for stem-like tumor cells in BC [137] and thus to chemoresistance of BCSC (Figure 2).

## 4. lncRNAs and Drug Resistance in Prostate Cancer

PCa is the most frequent malignant tumor in males and the second most common cause for cancer-related death, accounting for 10% of all cancer deaths. In 2019, roughly 174,650 patients were confronted with a PCa diagnosis in the USA [138]. Depending on clinico-pathological risk factors, standard therapy options for curative non-metastatic stages include watchful waiting for low-risk PCa or radical prostatectomy and radiotherapy for PCa with high-risk features. Approximately 4% of PCa cases are diagnosed with primary metastatic disease and several patients experience distant recurrence in treatments with curative intention [139]. Standard systemic therapy in palliative settings are based on androgen deprivation therapy (ADT) [140]. Yet, a significant proportion of patients eventually progress on ADT and develop castration resistant PCa (CRPC). This is due to androgen-independent signaling of the androgen receptor (AR) and consecutive androgen-independent downstream signaling [141]. Treatment options upon progression into CRPC include novel antiandrogens such as abiraterone [142] or enzalutamide [3] and chemotherapy regimens with docetaxel [143] and cabazitaxel [144]. Non-coding RNAs were reported to participate in PCa carcinogenesis [14,145,146]. Their potential involvement in the development of resistance to chemotherapy and ADT may pave the way for future therapeutic targets to overcome drug resistance in PCa.

### 4.1. lncRNAs Promoting Drug Resistance in PCa

#### 4.1.1. UCA1

**Figure 3:**
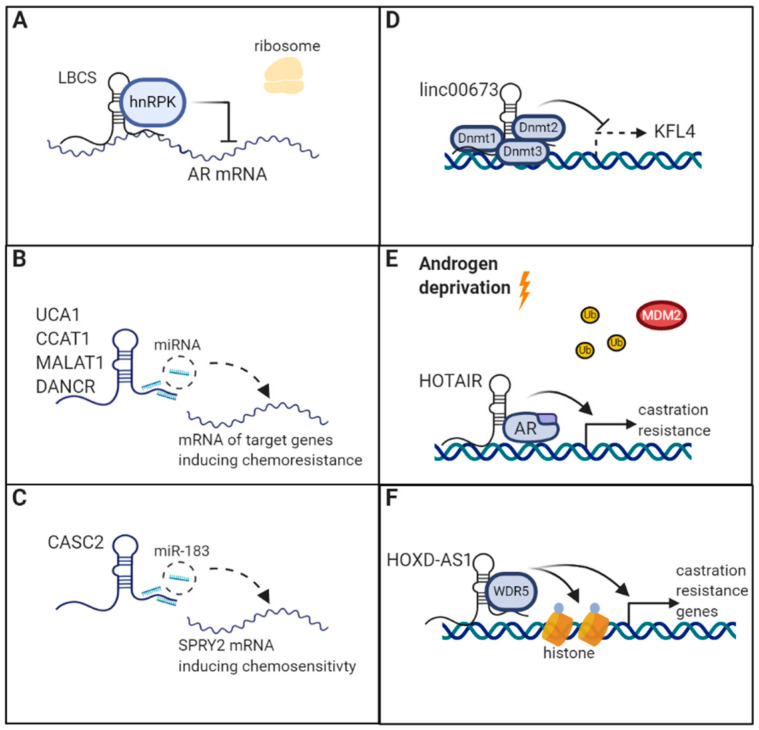
Examples of long non-coding RNAs (lncRNAs) and mechanisms of drug resistance in prostate cancer (PCa). (**A**) lncRNA low expression in bladder cancer stem cells (LBCS) recruits heterogeneous nuclear ribonucleoprotein K (hnRNPK) to the androgen receptor (AR) mRNA thereby preventing its translation. (**B**) lncRNAs sponge microRNAs and prevent them from binding to their targets. This leads to increased expression of the target proteins and induction of chemoresistance. (**C**) lncRNA cancer susceptibility candidate 2 (CASC2) acts as a competing endogenous RNA and sponges miR-183. This leads to increased expression of sprouty RTK signaling antagonist 2 (SPRY2) and restores resistance to chemotherapy. (**D**) lncRNA LINC00673 recruits DNMT1, DNMT22, and DNMT33 to the Krüppel-like factor 4 (KLF4) promoter and inhibits its transcription through increased methylation. This leads to increased chemoresistance. (**E**) lncRNA HOX transcript antisense RNA (HOTAIR) is increased under androgen deprivation and directly binds to the AR. This prevents AR ubiquitination and degradation by E3-ubiquitin-protein-ligase MDM2 and leads to AR signaling despite androgen deprivation therapy in castration resistant PCa. (**F**) lncRNA HOXD antisense RNA 1 (HOXD-AS1) recruits WD repeat-containing protein 5 (WDR5) to the promoter of chemo- and castration resistance-associated genes and mediates histone H3 lysine 4 tri-methylation (H3K4me3), thereby regulating the expression of target genes. (created with Biorender.com).

UCA1 plays a role in drug resistance across several cancer entities [147]. In PCa, UCA1 acts as a ceRNA and upregulation enhances tumor cell proliferation and progression [148,149]. Moreover, UCA1 may influence chemoresistance in PCa by sponging miR-204 (Figure 3B) [34]. Wang et al. [34] demonstrated the regulation of chemosensitivity via a UCA1/miR-204/Sirt1 pathway. Increased expression of UCA1 leads to the downregulation of miR-204 levels, resulting in elevated expression of Sirt1, which represents a target of miR-204 [34]. Sirt1 was previously demonstrated to enhance chemoresistance in PCa, corroborating this result [150]. Interestingly, elevated levels of UCA1 and Sirt1 and accordingly reduced levels of miR-204 were found in drug resistant PCa cancer cell lines, as compared to wildtype cell lines. Down- or upregulation of UCA1 or miR-204, respectively, improved docetaxel sensitivity and negatively influenced the expression of P-glycoprotein, a membrane pump that plays a major role in chemoresistance [34,151].

#### 4.1.2. MALAT1

lncRNA MALAT1 facilitates resistance to docetaxel in PCa through a MALAT1/miR-145-5p/AKAP12 axis, as proposed by Xue et al. [36], who demonstrated MALAT1 and A-kinase anchoring protein 12 (AKAP12) competing for miR-145-5p. Consequently, overexpression of MALAT1 leads to increased docetaxel resistance in DU145 and PC3 PCa cell lines, both in in vitro and in vivo models. MALAT1 levels were also demonstrated to be significantly increased in docetaxel-resistant cells as compared to chemosensitive cells [36]. This corroborates previous results of altered MALAT1 expression in cancer [152,153]. Furthermore, MALAT1 was found to be involved in PCa invasion, proliferation and progression [154,155,156]. However, MALAT1 may also play a part in CRPC and in the pathogenesis of enzalutamide resistance [37], Enzalutamide is a second-generation AR antagonist that significantly improves outcomes after failure of primary ADT [3,157]. Wang et al. [37] found that MALAT1 mediated enzalutamide resistance, as it is a regulator of AR-v7 (also called AR3). AR-v7 is the most abundant splicing variant of AR and strongly associated to enzalutamide resistance in CRPC [158]. Mechanistically, overexpressed MALAT1 may influence enzalutamide resistance by forming a complex with the pre-mRNA splicing factor SF2 and thereby promoting its activity and positively regulating AR-v7 splicing. The suppression of MALAT1 as a potential therapeutic approach to overcome resistance to enzalutamide was shown in vitro and in vivo, respectively [37]. Interestingly, MALAT1 expression levels were higher in circulating tumor cells (CTC) of CRPC patients and analysis of the TCGA dataset revealed adverse prognosis of PCa patients with MALAT1 overexpression [37].

#### 4.1.3. LINC00673

lncRNA LINC00673, which was found to be overexpressed in PCa, may affect drug resistance by recruiting DNA-methyltransferases (DNMT1, DNMT3a, and DNMT3b) to the KLF4 promoter. This results in enhanced methylation and epigenetic regulation of KLF4 expression (Figure 3D) [38]. KFL4 is involved in the regulation of many cellular functions such as cell growth, proliferation and differentiation, and may act as both a tumor suppressor and oncogene in a cellular context-dependent manner. In cancer, KLF4 function is frequently lost due to hypermethylation of CpG islands in the promotor region [159]. Suppression of LINC00673 led to improved chemosensitivity through increased KLF4 activity in both in vitro and in vivo experimental models and additionally reduced proliferation in PCa cell lines [38]. This is in line with results in gastric cancer, where epigenetic suppression of KLF4 through LINC00673 was associated with poor OS (HR = 2.989, 95%CI 1.126–5.178, *p* = 0.001) [160].

#### 4.1.4. LINC00518

Increased levels of LINC00518 were found in both PCa cell lines and in PCa tumor tissues [39]. However, even higher expression levels were reported in chemoresistant PCa cell lines and PCa patients who were resistant to paclitaxel treatment and LINC00518 was related to poor outcome in a study by He et al. [39], that enrolled 45 patients. Missing uni- and multivariate analyses should be considered as a limitation. The authors proposed LINC00518 to enhance chemoresistance through a LINC00518/miR-216b-5p/GATA6 pathway. The sponging of mir-216-5p with LINC00518 prevents it from binding to its target, the transcription factor GATA6 [39]. GATA6 is involved in the pathogenesis and progression of several cancer entities [161]. Interestingly, miR-216b-5p additionally appears to play a role in drug resistance of other malignancies, such as melanoma and NSCLC, however, other target proteins were allocated to this effect [100,162]. Nonetheless, this supports the results by He et al. [39].

#### 4.1.5. CCAT1

The colon cancer-associated transcript 1 (CCAT1) is an oncogenic lncRNA which has been widely reported to participate in cancer development and its utility as a biomarker in colorectal cancer has been demonstrated [163,164]. Accordingly, CCAT1 was demonstrated to be upregulated in PCa and was shown to enhance PCa proliferation, migration and invasion [40,165,166]. Only recently has the regulation of CCAT1 in metastatic CRPC via Vir-like m6A methyltransferase-associated protein (VIRMA)-dependent RNA-methylation become better understood [167]. In addition, Li et al. [40] identified CCAT1 as a potential novel target to influence drug resistance in PCa. Mechanistically, CCAT1 sequesters miR-24-3p and prevents it from targeting its downstream target fascin actin-bundling protein 1 (FSCN1), leading to increased FSCN1 expression levels and enhanced resistance to paclitaxel. Previous reports support the role of FSCN1 in chemoresistance [168,169].

#### 4.1.6. DANCR

The lncRNA differentiation antagonizing non-protein coding RNA (DANCR) affects chemoresistance of PCa cells by acting as a ceRNA, in this case for miR-34a-5p. miR-34a-5p was identified to directly bind to the 3′UTR of JAG1 (Jagged 1) mRNA leading to a reduction in JAGI expression levels [41]. Both, DANCR and JAG1 were found to be overexpressed in both PCa tissues of docetaxel-resistant patients and the docetaxel-resistant PCa cell lines DU-145 and PC-3. Silencing of DANCR could restore sensitivity to docetaxel treatment in vitro through the proposed DANCR/miR-34-5p/JAG1 axis, resulting in lower expression of P-glycoprotein and MRP1 and LRP1 proteins, which are associated with drug resistance in cancer [41]. JAG1 is part of the Notch signaling pathway and has been associated with disease progression and poor outcome in various cancer entities [170]. Interestingly, both JAG1 and miR-34-5p have already been demonstrated to influence drug resistance in other cancer entities [171,172], supporting the results of Ma et al. [41].

#### 4.1.7. HOXD-AS1/HAGLR

The lncRNA HOXD antisense RNA 1 (HOXD-AS1), also called HAGLR (HOXD antisense growth-associated long non-coding RNA) is strongly related to cancer [173]. It is overexpressed in CRPC cell lines and its expression significantly associates with adverse prognostic clinico-pathological biomarkers such as Gleason-score, T-stage and nodal invasion [42]. In fact, increased HOXD-AS1 levels were significantly associated with poor PFS (HR 2.827, 95%CI 1.297–6.161, *p* = 0.009) in an analysis that included 309 PCa patients from the TCGA database [42]. Moreover, besides promoting cell proliferation, HOXD-AS1 enhances castration resistance to bicalutamide therapy as well as chemoresistance to paclitaxel in vitro and in vivo [42]. Mechanistically, HOXD-AS1 recruits WD repeat-containing protein 5 (WDR5), thereby mediating histone H3 lysine 4 tri-methylation (H3K4me3) (Figure 3F) [42,174]. This results in activated transcription of castration and chemoresistance, as well as proliferation-associated genes including PKL1, AURKA, FXM1, CDC25C, UVE2C, CCNA2, and CCNB1 [42]. Gu et al. [42] conclude that HOXD-AS1 may represent a future therapeutic target to reinstall castration- and chemosensitivity.

#### 4.1.8. FEZF1-AS1

lncRNA FEZ family zinc finger 1—antisense RNA 1 (FEZF1-AS1) is an oncogenic lncRNA that is upregulated among various human malignancies. Its relation to tumor proliferation, migration and invasion, as well as its involvement in EMT through influencing tumorigenesis-associated pathways such as STATA3 and Wnt/β-catenin, has been demonstrated [175]. In PCa, FEZF1-AS1 regulates resistance to paclitaxel via the FEZF1-AS1/miR-25-3p/ITGB8 pathway [43]. By sponging miR-25-3p, which directly targets ITGB8, FEZF1-AS1 prevents degradation of ITGB8 mRNA, resulting in preserved influence on chemoresistance as well as cell viability, EMT, and cell autophagy [43]. Wang et al. [43] demonstrated that silencing of FEZF1-AS1 could restore sensitivity to paclitaxel both in vitro and in vivo. ITGB8 has been shown to alter treatment resistance in several cancer entities, including ovarian and hepatic cancers and glioblastoma [176,177,178].

#### 4.1.9. HOTTIP

The lncRNA HOXA distal transcript antisense RNA (HOTTIP) is strongly connected to cancer and has previously been reported to enhance tumor progression and chemoresistance [179]. Jiang et al. [44] were the first to investigate HOTTIP’s involvement in PCa chemoresistance. As reported, suppression of HOTTIP resulted in increased cisplatin sensitivity in the PCa cell lines DU-145 and PC-3 by inhibiting Wnt/β-catenin signaling [44]. This corroborates a number of previous studies that found HOTTIP to promote chemoresistance in numerous cancer entities [180,181,182], including osteosarcoma in which the regulation of Wnt/β-catenin by HOTTIP was also described [182]. However, Jiang et al. [44] did not investigate if other potential effectors such as miRNAs or proteins are involved in the connection between HOTTIP and Wnt/β-catenin regulation and moreover in vivo models are missing.

#### 4.1.10. PCGEM1

PCGEM1 (prostate cancer gene expression marker 1) may regulate AR splicing and expression of AR3 (AR-v7) [45], and therefore participate in the development of enzalutamide resistance [158]. Mechanistically, in a recent study by Zhang et al. [45] PCGEM1 expression is promoted by androgen deprivation, leading to intracellular transfer in nuclear speckles. Furthermore, PCGEM1 regulates the activity of the splicing factors nhRNP A1 and U2AF65, which are competing for AR3 splicing. This results in suppression (mediated through nhRNP A1) or enhancement (mediated through U2AF65) of AR3 expression by alternative splicing. [45]. Nevertheless, available data is conflicting. Parolina et al. [183] report opposite results as PCGEM1 is downregulated under androgen deprivation and no nuclear speckles were formed. In conclusion, the role of PCGEM1 in PCa and AR-regulation is not yet conclusively defined, considering several studies showing inconsistent results [45,183,184,185].

#### 4.1.11. HOTAIR

The lncRNA HOX transcript antisense RNA (HOTAIR) is upregulated in CRPC and may impact the development of enzalutamide resistance [46]. HOTAIR is suppressed by androgen through the AR protein and therefore upregulated under androgen deprivation and in CRPC. Moreover, increased HOTAIR expression was associated with shorter survival in a Kaplan–Meier analysis of two publicly available databases (*p* = 0.04, no uni- and multivariate models conducted) [46]. However, under androgen deprivation, HOTAIR expression increases and directly interacts with the AR protein at its N-terminal end and subsequently prevents ubiquitination and consequent degradation by the E3-ubiquitin-protein-ligase MDM2. This preserves AR-signaling and transcriptional activity, thereby leading to castration resistance (Figure 3E). In a cell culture model of enzalutamide resistant cells, HOTAIR expression steadily increased over the time as cells were exposed to enzalutamide treatment, indicating its indispensable role in developing resistance to enzalutamide [46].

#### 4.1.12. LBCS

The lncRNA LBCS was only recently described to exert regulatory function of AR-signaling in CRPC [47]. Specifically, LBCS expression is decreased in CRPC cell lines models and decreased expression was significantly and independently associated with shorter biochemical recurrence-free survival (BRFS) (HR = 0.447, 95%CI = 0.235–0.967, *p* = 0.040). It was demonstrated that induced overexpression of LBCS could reinstall sensitivity to androgen deprivation by recruiting hnRNPK binding directly to the AR mRNA and thereby limiting AR translation and activation [47] (Figure 3A). The mechanism of hnRNPK involvement in AR regulation has been reported previously [186].

### 4.2. lncRNAs Enhancing Drug Sensitivity in PCa

#### CASC2

Another lncRNA connected to docetaxel resistance in PCa cell lines is cancer susceptibility candidate 2 (CASC2) which has been associated with chemoresistance across various cancer entities, including gastric cancer [187], glioma [188], breast cancer [189], cervical cancer [190] and NSCLC [191]. In PCa, higher expression levels of CASC2 prevented miR-183 from binding to the 3′UTR of sprouty RTK signaling antagonist 2 (SPRY2) by acting as a ceRNA, thus preserving SPRY2 expression and inhibiting the downstream ERK signaling pathway, which is linked to chemoresistance [35,192] (Figure 3C). Activation of the ERK pathway in chemoresistant PCa cell lines was confirmed by a recent study investigating chemoresistance in PCa [40]. As a result, CASC2 promoted sensitivity to docetaxel in PCa cells [35].

## 5. lncRNAs and Drug Resistance in Testicular Cancer

TC accounts for 1% of cancers in males, which is approximately 55,000 patients per year worldwide [1,193]. Germ cell tumors are responsible for 95% of TC cases and can be subdivided into seminomas and non-seminomas [194].

Treatment strategies for localized TC include surgery (hemicastration or castration) as well as adjuvant chemotherapy depending on the presence of risk factors such as tumor extension and tumor marker decline (AFP, βhCG, LDH) after surgery, which classifies patients in good, intermediate and poor risk groups [195]. As for systemic therapy options, standard-of-care in the adjuvant as well as first-line metastatic setting is cisplatin-based chemotherapy [196].

In TC, noncoding RNAs have already been investigated in regards to their involvement in pathogenesis and their potential utility as clinical biomarkers [197,198]. The fact that patients with TC show great response to cisplatin-based chemotherapy may be a reason why data on lncRNAs in the development of chemoresistance is extremely limited. In fact, to the best of our knowledge, there is only one study that investigated this subject [48].

### H19

H19 is a well-investigated oncogenic lncRNA across various cancer types [199]. Regarding its involvement in chemoresistance in seminoma, Wei et al. [48] found H19 expression to be increased in the tissues of seminoma patients who were resistant to cisplatin-based chemotherapy, as well as in a cisplatin-resistant seminoma cell line. By acting as a ceRNA and thereby sponging miR-106-5p, which targets testis development related 1 (TDRG1), overexpression of H19 results in enhanced TDRG1 expression and reduced sensitivity to cisplatin treatment [48]. The authors proposed TDRG1, whose expression is exclusive in testis, to influence chemoresistance based on a previous work, which reported TDRG1 affecting resistance to cisplatin treatment in seminoma by regulating the PI3K/Akt/mTOR pathway [48,200,201]. Wei et al. [48] validated the promotion of chemoresistance by H19 in additional in vivo experiments, however, they only used one single cell line throughout their study and tissue samples of only 10 patients were used. Therefore, the results must be interpreted with caution and should only be considered hypothesis-generating until confirmed by further research. H19 was found to impact cisplatin resistance in other cancer entities [202,203,204,205], which is in line with and may support the results by Wei et al. [48].

## 6. Conclusions

In this review, we gave a comprehensive overview of lncRNAs and their involvement in drug resistance in cancers of the genitourinary system. Understanding the relation between lncRNAs and the biomarkers use and the development of drug and therapy resistance is crucial as lncRNAs may represent powerful therapeutic targets to overcome drug resistance and improve the outcome of cancer patients [206,207,208,209].

## Figures and Tables

**Table 1:** Long non-coding RNAs (lncRNAs) associated with drug resistance in cancers of the genitourinary system: Abbreviations: RCC- renal cell carcinoma, OS—overall survival, PFS—progression free survival, DFS—disease-free survival, BRFS—biochemical recurrence free survival, NA—not applicable.

lncRNA	Drug Resistance	Influence on Resistance ↑/↓	Expression Pattern	Pathway	Patient Tissue	In Vivo Models	Clinical Endpoint	Outcome (High Expression)	Cohort Size	Database	Multivariate Analysis	Hazard Ratio (HR) (95%CI, *p*-Value)	Ref
**RCC**													
SRLR	Sorafenib	↑	↑	SRLR/ NF-_Κ_B/ IL-6/ STATA3	Yes	Yes	PFS, treatment response	Poor	161	Institutional	Yes	PFS: 0.407 (0.222–0.744, *p* = 0.003)	[15]
ARSR	Sunitinib	↑	↑	ARSR/ miR-34/ AXL ARSR/ miR-34/ c-MET	Yes	Yes	PFS, treatment response	Poor	84	Institutional	Yes	PFS: 2.9, (1.2–7.1, *p* = 0.017)	[16]
ADAMTS9-AS2	5-fluorouracile, Cisplatin	↓	↓	ADAMTS9-AS2/ miR-27-3p/ FOXO1	yes	No	OS, DFS	Good	258 76	GEIPA Institutional	No	NA	[17]
NEAT1	Sorafenib	↑	↑	NEAT1/ miR-34a/ c-MET	Yes	No	OS, PFS	Poor	102	Institutional	No	NA	[18]
GAS5	Sorafenib	↓	↓	GAS5/ miR-21/ SOX5	Yes	Yes	No	NA	NA	NA	NA	NA	[19]
**Bladder cancer**													
UCA1	Cisplatin/gemcitabine	↑	↑	UCA1/ CREB/ miR-196a-5p/p27^Kip1^	Yes	Yes	No	NA	NA	NA	NA	NA	[20]
	Cisplatin	↑	↑	UCA1/Wnt6/Wnt signaling	Yes	Yes	No	NA	NA	NA	NA	NA	[21]
	NA	↑	↑	UCA1/miR-582-5p/ATG7-autophagy	Yes	Yes	No	NA	NA	NA	NA	NA	[22]
TUG1	Cisplatin	↑	↑	TUG1/miR-194-5p/CCND2	Yes	Yes	OS	Poor	87	Institutional	No	NA	[23]
PVT1	Doxorubicin/ cisplatin	↑	↑	PVT1/ Wnt/ β-catenin	Yes	No	No	NA	NA	Institutional	NA	NA	[24]
FOXD2-AS1	Gemcitabine	↑	↑	FOXD2-AS1/miR-143/ABCC3	No	Yes	No	NA	NA	NA	NA	NA	[25]
DLEU1	Cisplatin	↑	↑	DLEU1/miR-99b/HS3ST3B1	Yes	No	OS	Poor	406 485	TCGA (UALCAN/ KMplotter)	No	OS: 1.65 (1.2–2.26) *p* = 0.0016	[26]
MST1P2	Cisplatin	↑	↑	MST1P2/ miR-133b/ Sirt1/p53	No	No	No	NA	NA	NA	NA	NA	[27]
HIF1A-AS2	Cisplatin	↑	↑	HIF1A-AS2/HMGA1/p53 family	Yes	No	No	NA	NA	NA	NA	NA	[28]
GHET1	Gemcitabine	↑	↑	GHET1/ABCC1	Yes	No	No	NA	NA	NA	NA	NA	[29]
MALAT1	Cisplatin	↑	↑	MALAT1/miR-101-3p/ VEGF-C	Yes	No	No	NA	NA	NA	NA	NA	[30]
lncRNA-LET	Gemcitabine	↓	↓	LncRNA-LET/NF90/miR-145	Yes	Yes	OS	Good	60	Institutional	No	*p* = 0.0014	[31]
GAS5	Doxorubicin	↓	↓	GAS5/ Bcl-2	Yes	No	OS	Good	82	Institutional	No	OS: 0.4824 (0.2865–0.8122 *p* = 0.006)	[32]
LBCS	Cisplatin/gemcitabine	↓	↓	lnc-LBCS/ hnRNPK/EZH2/ SOX2	Yes	Yes	OS, DFS	Good	120/185	Institutional TCGA-GEPIA	Yes	OS: 0.2721, *p* < 0.0001 DFS: 0.3029 *p* < 0.0001 OS: 0.3663 *p* = 0.0165	[33]
**Prostate cancer**													
UCA1	Docetaxel	↑	↑	UCA1/ miR-204 /Sirt1	No	No	No	NA	NA	NA	NA	NA	[34]
CASC2	Docetaxel	↓	↓	CASC2/miR-183/SPRY2	Yes	No	No	NA	NA	NA	NA	NA	[35]
MALAT1	Docetaxel	↑	↑	MALAT1/miR-145-5p/AKAP12	Yes	Yes	No	NA	NA	NA	NA	NA	[36]
	Enzalutamide	↑	↑	MALAT1/SF2/AR-v7	No	Yes	No	NA	NA	NA	NA	NA	[37]
Linc00673	Paclitaxel Docetaxel	↑	↑	Linc00673/Dnmt1-3/KFL4	Yes	Yes	No	NA	NA	NA	NA	NA	[38]
Linc00518	Paclitaxel	↑	↑	Linc00518/miR-216-5p/GATA6	Yes	No	OS	Poor	45	Institutional	No	NA	[39]
CCAT1	Paclitaxel	↑	↑	CCAT1/miR-24-3p/FSCN1	Yes	No	No	NA	NA	NA	NA	NA	[40]
DANCR	Docetaxel	↑	↑	DANCR/miR-24a-5p/JAG1	Yes	Yes	No	NA	NA	NA	NA	NA	[41]
HOXD-AS1	Bicalutamide Paclitaxel	↑	↑	HOXD-AS1/WDR5	No	Yes	PFS	Poor	309	TCGA	Yes	2.827, (1.297–6.161) *p* = 0.009	[42]
FEZF1-AS1	Paclitaxel	↑	↑	FEZF1-AS1/miR-25-3p/ITGB8	Yes	Yes	No	NA	NA	NA	NA	NA	[43]
HOTTIP	Cisplatin	↑	↑	HOTTIP/Wnt/β-catenin	Yes	No	No	NA	NA	NA	NA	NA	[44]
PCGEM1	Enzalutamide	↑	↑	PCGEM1/AR3-splicing	No	Yes	No	NA	NA	NA	NA	NA	[45]
HOTAIR	Enzalutamide	↑	↑	HOTAIR/AR	Yes	No	BRFS	Poor	NA	GEO	No	NA	[46]
LBCS	Bicalutamide	↓	↓	LBCS/ hnRNPK/AR	Yes	No	BRFS PFS	Good	374	TCGA	Yes	BRFS: 0.447 (0.235–0.967) *p* = 0.040	[47]
**Testicular Cancer**													
H19	Cisplatin	↑	↑	H19/miR-106-5p/TDRG1	Yes	Yes	No	NA	NA	NA	NA	NA	[48]
